# The Complete Chloroplast Genome and the Phylogenetic Analysis of *Panicum bisulcatum* (Thumb.) (Poaceae)

**DOI:** 10.3390/ijms27010135

**Published:** 2025-12-22

**Authors:** Yuan Gao, Yutong Cai, Huifeng Wang, Zhihui Tian, Zhaofeng Huang

**Affiliations:** 1 Eco-Environmental Protection Research Institute, Shanghai Academy of Agricultural Sciences, Shanghai 201403, China; gaoyuan@saas.sh.cn (Y.G.); 1476838839@nefu.edu.cn (Y.C.); 2 Key Laboratory of Low-Carbon Green Agriculture in Southeastern China, Ministry of Agriculture and Rural Affairs, Shanghai 201403, China; 3 School of Chemical and Environmental Engineering, Shanghai Institute of Technology, Shanghai 201418, China; hfwang@sit.edu.cn; 4 State Key Laboratory for Biology of Plant Diseases and Insect Pests, Institute of Plant Protection, Chinese Academy of Agricultural Sciences, Beijing 100193, China

**Keywords:** *Panicum bisulcatum*, chloroplast genome, phylogenomics, inverted repeat, single nucleotide polymorphism

## Abstract

The chloroplast (cp) genome of *Panicum bisulcatum* (Thumb.), a significant agricultural weed, was sequenced and characterized to elucidate its genomic architecture, evolutionary dynamics, and phylogenetic relationships. The complete cp genome was assembled as a circular DNA molecule of 138,489 bp, exhibiting a typical quadripartite structure comprising a large single-copy (LSC, 82,260 bp), a small single-copy (SSC, 12,569 bp), and a pair of inverted repeats (IR, 21,830 bp each) regions. It encodes 135 genes, including 89 protein-coding genes, 49 tRNAs, and 8 rRNAs. Functional annotation revealed that most genes are involved in photosynthesis and genetic system. A total of 51 simple sequence repeats (SSRs) and 62 long repeats (LRs) were identified, providing potential molecular markers. Comparative analysis of IR boundaries highlighted both conserved features and species-specific expansion/contraction events among *Panicum* species. Phylogenomic analysis robustly placed *P. bisulcatum* within the genus *Panicum*, showing a closest relationship with *P. incomtum* and confirming the monophyly of the genus. Furthermore, single nucleotide polymorphism (SNP) analysis with its closest relative, *P. incomtum*, revealed 4659 SNPs, with a dominance of synonymous substitutions, indicating the action of purifying selection. This study provides the first comprehensive cp genomic resource for *P. bisulcatum*, which will facilitate future studies in species identification, phylogenetic reconstruction, population genetics, and the development of sustainable management strategies for this weed.

## 1. Introduction

The Poaceae family encompasses the world’s most vital cereal crops and numerous ecologically significant grasses, but also many species that have adapted to become persistent and economically damaging weeds [1,2]. The genus *Panicum* (tribe Paniceae, subfamily Panicoideae) is particularly noteworthy due to its remarkable diversity in photosynthetic pathways, life histories, and ecological niches. *Panicum bisulcatum* (Thunb.), a diploid (2n = 2x = 18) annual grass, represents an early-diverging C_3_ lineage within this largely C_4_-dominated tribe [3,4]. Naturally distributed across temperate and tropical East Asia, it commonly inhabits open, disturbed, and moist environments [5]. In recent decades, *P. bisulcatum* has emerged as a problematic weed in rice paddies, owing to its synchronized phenology, strong competitive ability, and prolific seed production that builds a persistent soil seed bank [6,7,8,9]. Its adaptability to waterlogged conditions further complicates management [6,7], leading to substantial yield losses if uncontrolled [8]. Understanding the genetic basis of its adaptability is therefore crucial for developing sustainable integrated weed management strategies.

Advances in high-throughput sequencing have revolutionized plant systematics and evolution, positioning the chloroplast (cp) genome as a key molecular marker [10,11,12]. The typical angiosperm cp genome is a circular, quadripartite DNA molecule (120–160 kbp) consisting of a large single-copy (LSC) region, a small single-copy (SSC) region, and two inverted repeat (IR) regions [13,14,15]. It is generally maternally inherited, non-recombining, and evolves at a relatively slow yet informative rate, making it ideal for resolving phylogenetic relationships at genus and species levels [16,17,18,19]. For complex genera like *Panicum*, robust phylogenies are essential to unravel evolutionary trajectories such as the multiple origins of C_4_ photosynthesis. While earlier studies using limited loci provided preliminary frameworks, they often lacked resolution for closely related species [3]. Complete cp genome sequences deliver a substantial increase in phylogenetic information, enabling stronger clade support. Although cp genomes are available for several economically important *Panicum* species (e.g., *P. miliaceum* [20] and *P. virgatum*), early-diverging C_3_ lineages like *P. bisulcatum* remain uncharacterized. Sequencing its complete cp genome fills this taxonomic gap, allowing precise phylogenetic placement within Panicoideae and insights into the evolutionary transition from C_3_ to C_4_ syndromes. Beyond sequence data, it also facilitates the study of structural evolution, including IR boundary dynamics, gene order conservation, and intron gain/loss [21,22,23,24].

The utility of complete cp genomes extends beyond deep phylogeny to recent evolutionary and population-level processes [25,26]. The haploid, non-recombining nature of chloroplast DNA makes it highly sensitive for phylogeographic studies, tracing migration routes and identifying genetic lineages [27,28,29]. For *P. bisulcatum*, key questions remain regarding its invasion dynamics in Asian paddies—whether it resulted from multiple local adaptations or human-mediated dispersal. High-resolution population genetic studies are needed, and the complete cp genome provides the ideal resource for developing such markers. It contains abundant polymorphic sites, including single nucleotide polymorphisms (SNPs) and chloroplast simple sequence repeats (cpSSRs), which offer superior discriminatory power compared to standard barcoding loci [30,31,32,33]. These markers can elucidate fine-scale population structure, gene flow, and invasion origins. From an applied perspective, this information supports predictive modeling, biosecurity tracing, and monitoring of herbicide-resistant biotypes. Comparative genomic analysis can also identify hypervariable regions for designing species-specific PCR assays, enabling early detection of *P. bisulcatum* in soil seed banks or agricultural produce—a critical tool for precision weed management [34,35,36].

To address these gaps, we sequenced, assembled, and annotated the complete chloroplast genome of *P. bisulcatum*. The primary objectives were to: (1) characterize its genomic structure, gene content, and repetitive elements; (2) investigate IR boundary evolution through comparative analysis; (3) determine its phylogenetic placement within *Panicum* using whole cp genome data; and (4) assess sequence divergence and evolutionary constraints via SNP analysis against its closest relative, *P. incomtum*. This work provides the first comprehensive cp genomic resource for *P. bisulcatum*, facilitating both fundamental studies on Panicoideae evolution and applied efforts in monitoring and managing this significant agricultural weed.

## 2. Results

### 2.1. Chloroplast Genome Component

**Figure 1:**
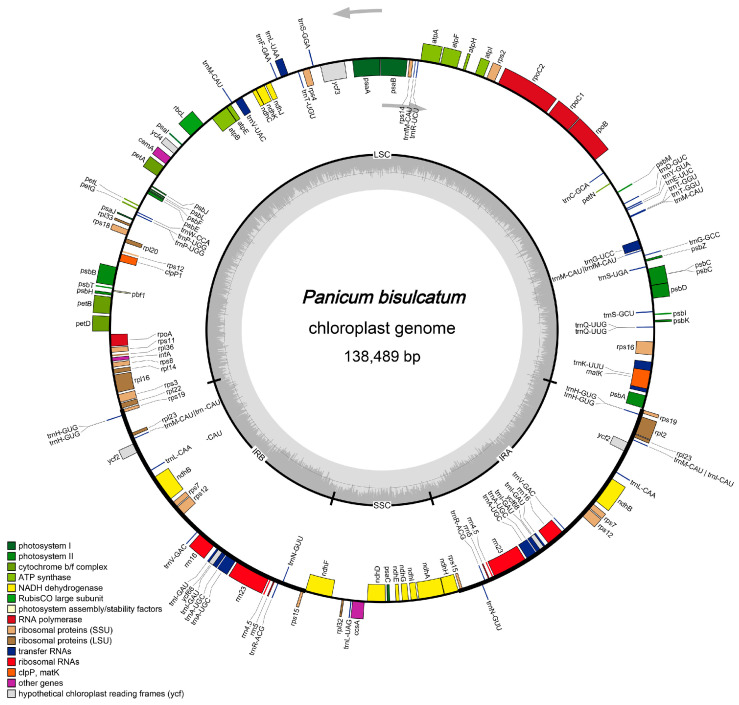
The assembly, size, and features of the chloroplast genome of *Panicum bisulcatum*. The arrows indicate the direction of transcription (clockwise for the + strand, counterclockwise for the − strand).

**Figure 2:**
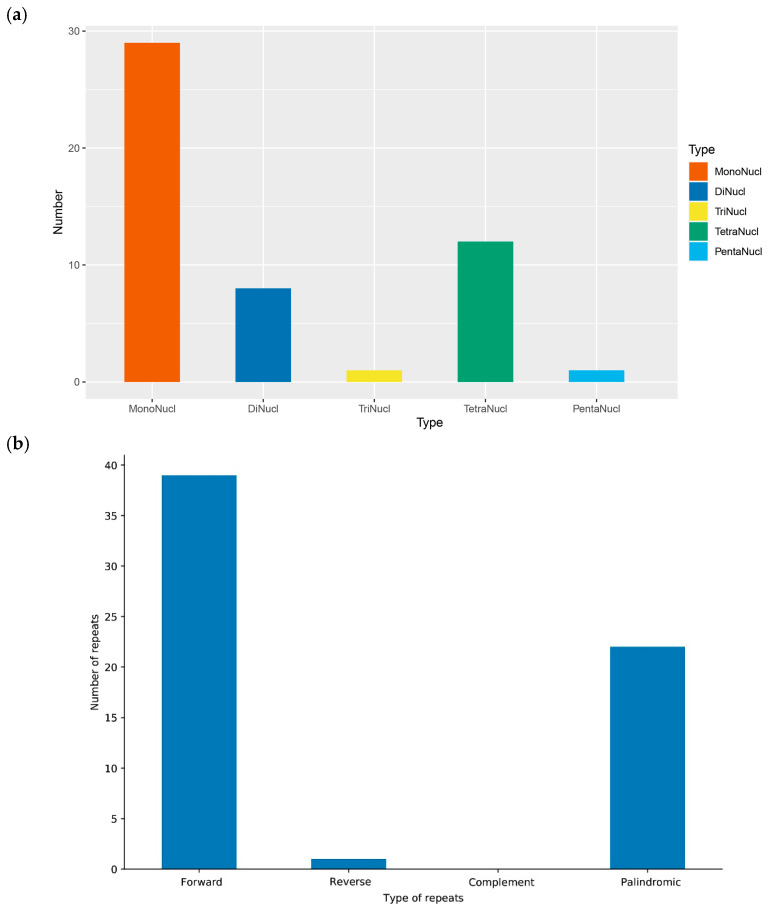
The repeat sequences in the chloroplast genome of *Panicum bisulcatum.* (**a**) Simple sequence repeats. (**b**) Long repeats.

The cp genome library of *Panicum bisulcatum* was constructed using the Illumina TruSeq™ Nano DNA Sample Prep Kit (San Diego, CA, USA). In total, after trimming low-quality fragments in the raw data, 32,612,668 clean reads were used for de novo assembly of the *P. bisulcatum* chloroplast genome using NOVOPlasty v4.2 software (https://github.com/ndierckx/NOVOPlasty, accessed on 7 April 2025), resulting in a circular genome of 138,489 bp (with 38.51% GC content). This sequencing has also reached sufficient sequencing depth (Supplementary Figure S1). The complete cp genomes displayed the typical quadripartite structure of most angiosperms, including LSC (82,260 bp), SSC (12,569 bp), and a pair of IRs (IRa and IRb) (21,830 bp) (Figure 1). The cp genome contained 135 genes, including 89 protein-coding genes (with a total length of 63,861 bp, 39.20% GC content), 49 transfer RNA (tRNA) genes, and 8 ribosomal RNAs (rRNA) genes (with a total length of 12,913 bp). Notably, gene duplication is common in the chloroplast genome. Beyond the expected doubling of genes within the IR regions (due to their inverted repeat nature), certain genes in the LSC and SSC regions—such as *rps16*, *psbC*, and *ndhA*—also appear in two or more copies. The gene counts provided above represent unique gene types; the total number of gene copies in the genome is higher. Repeat sequences were also detected in the cp genome of *P. bisulcatum*. A total of 51 simple sequence repeats (SSRs) were identified, including 12 distributed in coding region (Supplementary Table S1). According to the number of repeated bases, the type of SSR was shown in Figure 2a. There were 62 long repeats (LR) in the cp genome of *P. bisulcatum* (Supplementary Table S2). According to the Hamming Distance (HD), the type of LR was shown in Figure 2b.

### 2.2. Gene Function Annotation and Classification

**Figure 3:**
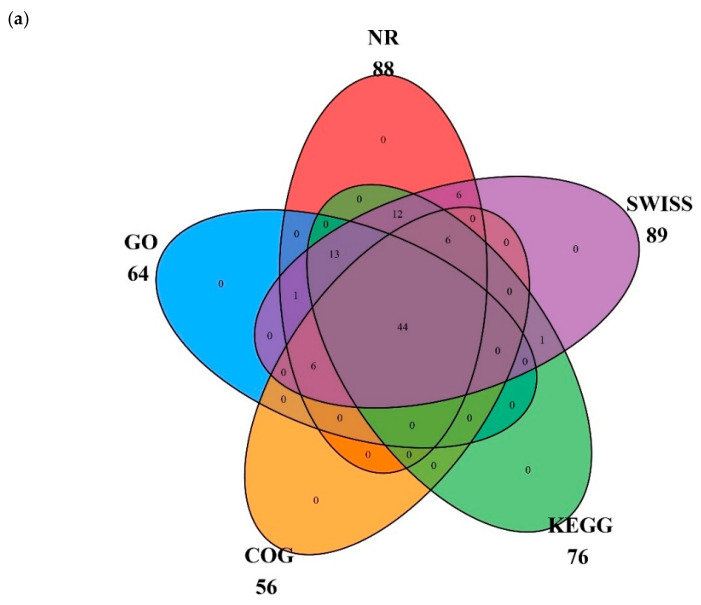

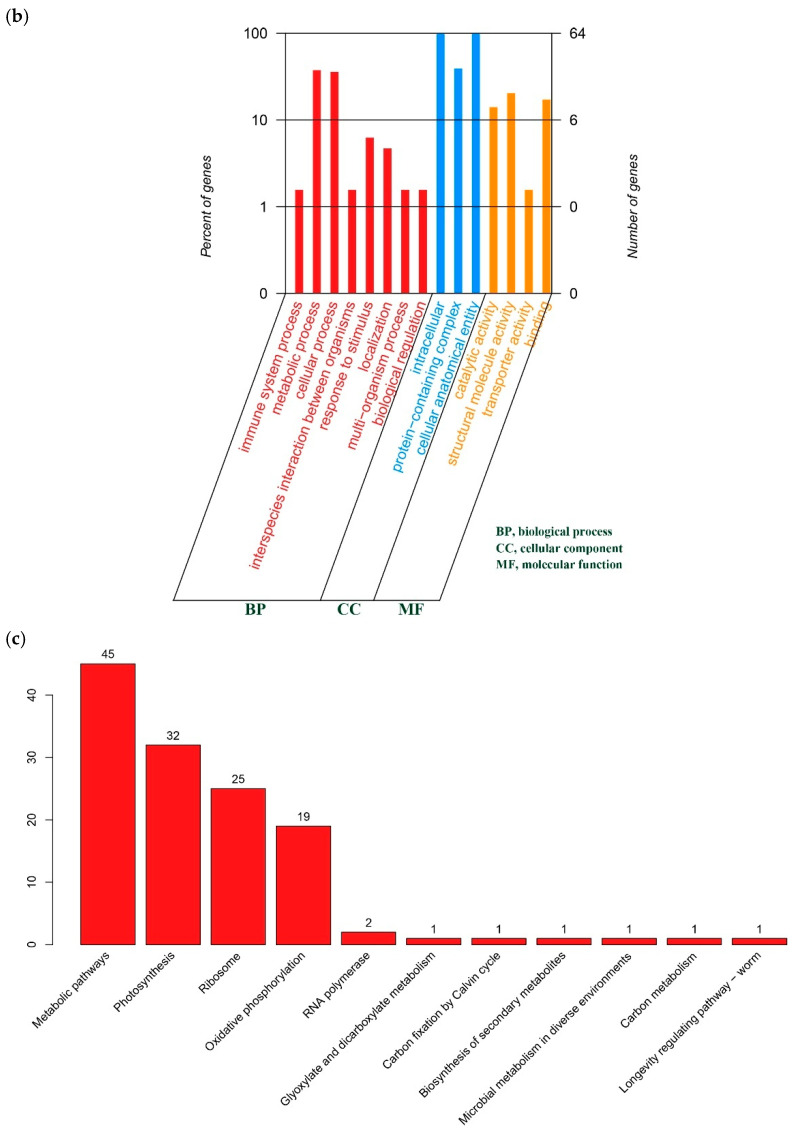
Classifications of genes function of *Panicum bisulcatum*. (**a**) Cross matching of coding genes to five databases. (**b**) Percentages of genes matched to GO function classification. BP means biological process, CC means cellular component, and MF means molecular function. (**c**) Top 11 of genes matched to KEGG pathways.

All genes in the cp genome of *Panicum bisulcatum* were functionally annotated and mainly belonged to the photosynthesis and genetic system categories, with 45 genes and 58 genes, respectively. The gene names, groups, and categories are listed in Table 1. In the *P. bisulcatum* cp genome, 88 genes were matched to Non-Redundant Protein Sequence Database (NR), 64 to Gene Ontology (GO), 56 to Clusters of Orthologous Groups (COG), 76 to Kyoto encyclopedia of Genes and Genomes (KEGG), and 89 to Swiss. Among these genes, 44 could be matched to all five databases; these cross-relationships are shown in Figure 3a. Genes matched to GO were further classified as biological process (BP), cellular component (CC), and molecular function (MF), with most genes classified as BP (Figure 3b). Genes matched to KEGG were mainly involved in metabolic pathways, photosynthesis, ribosome, and oxidative phosphorylation (Figure 3c).

Genes located in the Inverted Repeat regions are present in two copies.

### 2.3. Gene Order and Operon Organization

The chloroplast genome of *P. bisulcatum* exhibits a gene order and operon structure highly conserved across grasses. Key photosynthesis-related genes are organized into polycistronic units, such as the *psbB-psbT-psbH* operon within the LSC region (Figure 1, Table 1). Similarly, genes for the ATP synthase complex (*atpI-atpH-atpF-atpA*) and ribosomal proteins (e.g., *rps2*) are found in conserved clusters (Figure 1, Table 1). A prominent operon near the LSC/IRb boundary contains the genes *rps19*, *rpl22*, *rps3*, *rpl16* and so on (Figure 1, Table 1), which is a hallmark of grass chloroplast genomes. This operonic organization underscores the coordinated regulation of genes involved in related functions.

### 2.4. IR Expansion and Contraction

**Figure 4:**
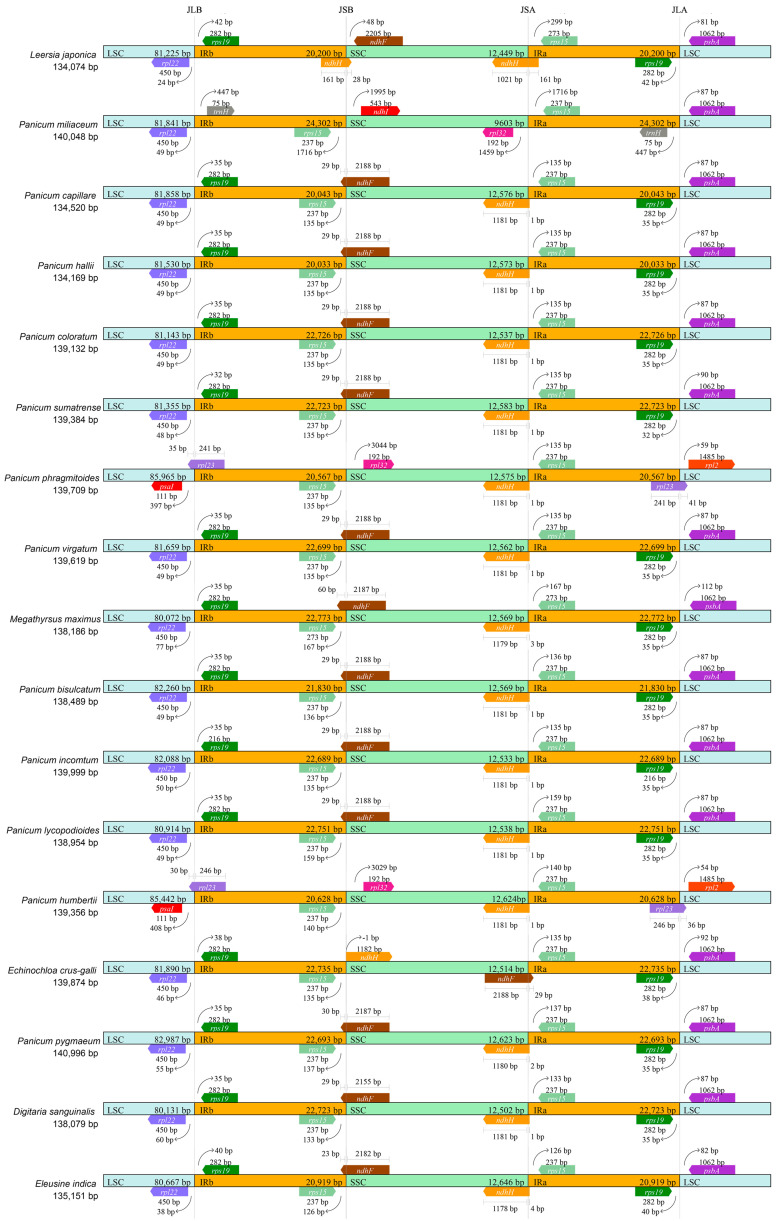
Comparison of large sequence copy (LSC), inverted repeat (IRb, IRa), and small sequence copy (SSC) border regions of the chloroplast genomes of representative Poaceae plants.

To further observe the potential expansion and contraction of the IR regions, gene variations at the IR/SSC and IR/LSC boundary regions of 13 plants of the genus *Panicum* and four common Poaceae plants in paddy fields were compared (Figure 4). The *rpl22* gene and *rps19* gene, located at LSC and IRb, respectively, are the boundary genes in most plants, except for *P. humbertii* and *P. phragmitoides*. The *ndhF* gene typically spans the SSC/IRb boundary, as observed in most selected plant species, while minor variations exist in its total length and the proportions extending into each region. The species exhibiting distinct boundary-spanning or boundary-proximal genes at this locus are *P*. *miliaceum*, *Echinochloa crus-galli*, *Leersia japonica*, *Panicum humbertii*, and *Panicum phragmitoides*. The *ndhH* gene represents another boundary-spanning locus (SSC/IRa) in the IR region of *P*. *bisulcatum*, despite having merely one bp extending into the IRa segment. In *P. bisulcatum*, *ndhH* represents an additional boundary-spanning gene (SSC/IRa) within the IR region of the cp genome, despite having only one bp extending into IRa. Notably, this gene maintains a conserved presence within IRa across most other species, with lengths typically ranging from 1 to 4 bp. Regarding genes associated with this boundary, *E. crus-galli* and *P. miliaceum* exhibited the most pronounced structural divergence. At the LSC/IRa boundary, *psbA*, and *rps19* were conserved as the boundary-associated genes in *P*. *bisulcatum* and most selected species, with the exception of *P*. *miliaceum*, *P*. *humbertii*, and *P*. *phragmitoides*, which exhibited a divergent arrangement.

### 2.5. Phylogenetic Analysis Among Common Poaceae Plants

**Figure 5:**
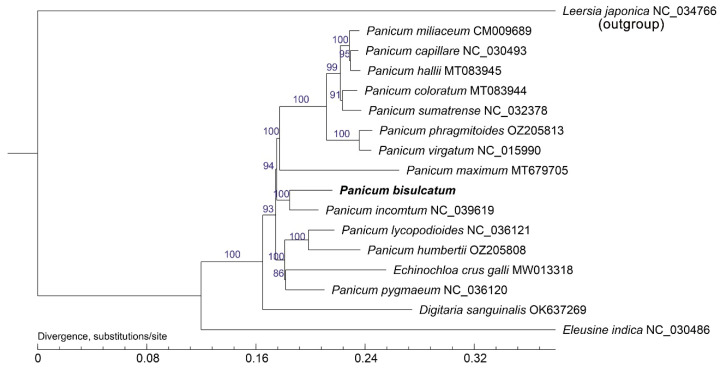
Phylogenetic and divergence analysis for 18 species of the family Poaceae. Phylogenetic trees using maximum likelihood (ML), based on alignments of complete chloroplast genomes. The tree was rooted using *Leersia japonica* as the outgroup.

The phylogenetic tree reconstructed from chloroplast genome data robustly resolves the phylogenetic position of *P*. *bisulcatum* within the genus *Panicum* (Figure 5). As illustrated, *P. bisulcatum* forms a well-supported clade with other *Panicum* species, confirming its taxonomic placement in the genus. Notably, it shares a most recent common ancestor with *P*. *miliaceum*, indicating a close evolutionary relationship between these two species. Overall, *Echinochloa crus-galli* and species of the genus *Panicum* are tightly clustered within a major clade, with *P*. *pygmaeum* sharing the most recent common ancestor with this group, strongly supporting a close phylogenetic affinity between these two genera. Within the broader context of the tree, the genus *Panicum*, which includes *P. bisulcatum*, is clearly delineated and exhibits a sister relationship with the genus *Digitaria* (e.g., *Digitaria sanguinalis*). This inter-tribal relationship receives strong nodal support, consistent with established classifications based on chloroplast genome. Additionally, based on the chloroplast genome data of the selected plant species, *Leersia japonica* is the most distantly related to *P. bisulcatum*, followed by *Eleusine indica*. *Panicum* species, including *P. bisulcatum*, form a distinct monophyletic group. This suggests evolutionary conservation within the genus. The overall topology and high bootstrap values at key nodes reinforce the reliability of the chloroplast genome in resolving phylogenetic relationships at both inter- and intra-generic levels.

### 2.6. Single Nucleotide Polymorphism Analysis

We performed single nucleotide polymorphisms (SNPs) analysis on the closest relative species, *Panicum incomtum*, using *P*. *bisulcatum* as the reference cp genome (Table 2). A total of 4659 SNPs were detected, highlighting a substantial degree of sequence divergence between these two species. These polymorphisms are distributed across both coding and non-coding regions, with 3281 SNPs located in coding sequences (CDS) and 1378 in intergenic regions. Within the protein-coding regions, the SNPs are categorized as either synonymous or nonsynonymous substitutions. The number of synonymous SNPs (2000) is notably higher than that of nonsynonymous SNPs (1252). This discrepancy suggests the presence of purifying selection, which acts to eliminate mutations that alter amino acid sequences, thereby preserving the functional integrity of essential chloroplast-encoded proteins. The prevalence of synonymous changes indicates that while the nucleotide sequence is evolving, the encoded proteins remain largely conserved. Furthermore, the significant number of intergenic SNPs underscores the higher evolutionary plasticity of non-functional regions compared to constrained coding sequences. Collectively, the SNP profile not only delineates clear genetic boundaries between *P. incomtum* and *P. bisulcatum* but also reflects the differential evolutionary pressures acting on various functional elements of the chloroplast genome.

## 3. Discussion

### 3.1. Genomic Architecture and Repetitive Elements of the P. bisulcatum Chloroplast Genome

Plants of the genus *Panicum* (Poaceae) are recognized as significant weed species in agricultural systems worldwide, often competing aggressively with crops and reducing yields [2,6,7,8]. In recent years, the spread and adaptive evolution of these weeds have posed increasing challenges to sustainable farm management, drawing greater research attention. *Panicum bisulcatum*, a representative species within the genus, has been frequently reported for its weedy characteristics, ecological adaptability, and in some cases, stress resistance [2,6]. Beyond its role as a weed, *P. bisulcatum*, like many congeneric species, may possess untapped practical value. Previous studies on related species have indicated potential allelopathic effects and bioactive properties, suggesting that plant extracts could be explored for use in natural pest control strategies or even for medicinal applications [37,38,39,40]. However, the chloroplast (cp) genome of *P. bisulcatum*—and indeed of many species within the genus—remains underexplored, creating a gap in our understanding of its evolution and relationships. Since the first cp genome of tobacco was sequenced [41], numerous plant lineages have been characterized, greatly facilitating phylogenetic and evolutionary studies [16,42,43,44,45]. As a widespread weed with potential utility, a detailed understanding of the cp genome architecture, informative repetitive sequences, and evolutionary context of *P. bisulcatum* is of both theoretical and practical importance.

In this study, we assembled and annotated the complete cp genome of *P. bisulcatum*. The genome spans 138,489 bp—a size consistent with other angiosperms in the Poaceae family [46]. It exhibits the highly conserved quadripartite structure, comprising a Large Single Copy (LSC: 82,260 bp), a Small Single Copy (SSC: 12,569 bp), and a pair of Inverted Repeats (IRs: 21,830 bp each). This structural conservation underscores the stability of the fundamental cp genome architecture across much of the plant kingdom [15,16,17,18,19,47,48]. A total of 135 genes were annotated, including 89 protein-coding genes, 49 tRNA genes, and 8 rRNA genes, with their distribution across the LSC, SSC, and IR regions following patterns observed in related grasses. Furthermore, we identified a total of 51 Simple Sequence Repeats (SSRs) and 62 Long Repeats (LRs). These repetitive elements are increasingly valued as molecular markers for population genetics, phylogeography, and species identification [33,49,50,51]. Their propensity to promote intermolecular recombination and slipped-strand mispairing also suggests they play a key role in generating genomic diversity and structural evolution [52,53,54]. The comprehensive SSR and LR profiles presented here provide a valuable foundation for future studies on genetic variation, population structure, and even the domestication history of *Panicum* species.

### 3.2. Gene Content and Functional Implications

Functional annotation confirmed that the majority of genes in the *P. bisulcatum* chloroplast genome belong to the conserved, core functional categories of photosynthesis and the genetic system, consistent with its essential roles in energy production and gene expression [55,56]. This conserved function is underpinned by a stable structural organization, including the presence of classic polycistronic operons (e.g., for photosystem II, ATP synthase, and ribosomal proteins) which facilitate coordinated transcriptional regulation and serve as valuable phylogenetic markers (Figure 1). Within this overall conserved framework, two notable features merit further discussion. First, while the total number of protein-coding genes is modest relative to some weedy relatives, key genes critical for function and resilience—such as *clpP1*, essential for development and stress response—are retained without loss [57,58]. Second, and more distinctive, is the detection of multiple copies of specific genes (e.g., *rps16*, *psbC*, *ndhA*) located outside the inverted repeat regions. These non-IR duplications, potentially originating from unequal homologous recombination, may provide a selective advantage through increased gene dosage for photosynthesis and translation or represent early stages of functional divergence. Together, the conserved operonic architecture, retention of key genes, and lineage-specific gene duplications illustrate a genome that balances evolutionary stability with localized plasticity. These characteristics likely contribute to the efficient gene expression and adaptive potential that support the ecological success of *P. bisulcatum*.

### 3.3. Comparative Analysis of IR Boundary Dynamics

A particularly dynamic aspect of chloroplast genome evolution involves the expansion and contraction of the Inverted Repeat (IR) regions, which primarily occurs at the junctions with the Single Copy (SC) regions [14,24,59]. Our comparative analysis of IR/SC boundaries among *Panicum* species and related genera revealed a mix of conserved and divergent features. Genes such as *rpl22* and *rps19* were conserved as boundary-associated genes across most species examined. However, we also observed lineage-specific patterns. For instance, the *ndhF* gene was found to span the IRb/SSC boundary in most species, including *P. bisulcatum*, while the *ndhH* gene was located at the SSC/IRa boundary with only a minimal extension into the IRa region. Such subtle variations in gene boundaries and the lengths of genes extending into the IR regions reflect the ongoing dynamic process of IR evolution. The unique arrangements observed in certain species, such as *P. miliaceum* and *P. humbertii*, underscore the evolutionary plasticity of these genomic regions. These structural variations are not merely neutral characteristics; they can influence gene dosage, affect genome stability, and potentially contribute to the generation of structural novelties, representing a crucial mechanism in plant chloroplast genome evolution.

### 3.4. Phylogenetic Position and Evolutionary Insights

Organelle-based barcodes derived from chloroplast genome data offer a powerful tool for species definition by resolving phylogenetic connections [60]. To unequivocally determine the phylogenetic position of *P. bisulcatum*, we reconstructed a phylogenetic tree using complete chloroplast genome sequences. The analysis robustly placed *P. bisulcatum* within the genus *Panicum*, showing a particularly close evolutionary relationship with *P. incomtum*. It is of particular significance, especially considering our previous finding that *Sesbania cannabina*—which is closely related to weeds in dryland rice fields, *Aeschynomene indica*—has also evolved into a paddy weed [45]. This serves as a crucial warning for weed invasion into cultivated ecosystems. The overall tree topology strongly supports the monophyly of *Panicum* and illustrates a clear sister relationship with the genus *Digitaria* (e.g., *Digitaria sanguinalis*), consistent with modern taxonomic classifications based on genomic data. Divergence time estimation further revealed that the split between *P. bisulcatum* and its closest relatives occurred millions of years ago, highlighting a long and independent evolutionary history within the genus. These results affirm the high resolution and utility of complete chloroplast genomes in resolving phylogenetic relationships at both inter- and intra-generic levels, providing a reliable evolutionary framework for future studies on the biogeography and trait evolution in this group [26,61].

### 3.5. Sequence Divergence and Evolutionary Constraints Revealed by SNP Analysis

Beyond structural and phylogenetic insights, we delved into the sequence-level divergence between *P. bisulcatum* and its close relative *P. incomtum* through Single Nucleotide Polymorphism (SNP) analysis. A substantial number of polymorphisms (4659 SNPs) were detected, highlighting a clear genetic distinction between the two species. The distribution of these SNPs was informative: a majority were located in coding sequences (CDS), and among these, synonymous substitutions significantly outnumbered nonsynonymous substitutions. This pattern is a classic signature of purifying selection, which acts to preserve the amino acid sequence and thus the functional integrity of essential chloroplast-encoded proteins [62,63]. The abundance of intergenic SNPs further indicates that non-functional regions evolve more rapidly, free from such strong selective constraints. This SNP profile not only provides a powerful set of molecular markers for distinguishing these species but also reflects the differential evolutionary pressures acting on various functional elements of the chloroplast genome [64,65].

### 3.6. Future Perspectives: Gene Transfer and RNA Editing

Beyond the structural and sequence-level analyses presented, the evolution of the chloroplast genome is also shaped by intracellular gene transfer, primarily from the chloroplast to the nuclear genome [66]. These transferred sequences, known as nuclear plastid DNAs (numts), can complicate phylogenetic analysis but also serve as markers of deep evolutionary history. While a systematic investigation of numts across *Panicum* requires comparative analysis of both nuclear and chloroplast genomes—a promising avenue for future research—the conserved nature of certain chloroplast-encoded genes (e.g., *clpP1*) and the integrity of the IR-boundary genes in *P. bisulcatum* provide a baseline for such studies. Future sequencing of nuclear genomes for *Panicum* species will enable the exploration of these transfer events and their role in the evolutionary integration of organellar and nuclear genetic material [48,66].

Beyond sequence and structural variations, post-transcriptional RNA editing represents another important dimension of chloroplast genome evolution and regulation, as noted in previous studies [67,68]. This process, primarily involving C-to-U conversions, can alter amino acid sequences and potentially influence the function, stability, or assembly of protein complexes such as the NADH dehydrogenase and photosystems. While the prediction of editing sites in silico is possible, their functional validation requires transcriptome data. In the context of *P. bisulcatum*, a systematic characterization of its chloroplast RNA editome—and its comparison with other *Panicum* species—remains an open and valuable question for future research. Such comparative studies could reveal whether specific editing patterns are associated with environmental adaptation or the evolutionary divergence between C_3_ and C_4_ lineages within the genus.

## 4. Materials and Methods

### 4.1. DNA Extraction and Sequencing

Fresh leaves and stems were harvested from *Panicum bisulcatum* from paddy field in Shanghai (31°27′21″ N, 121°52′58″ E), China, in 2024. Total genomic DNA was extracted following a modified cetyltrimethylammonium bromide (CTAB) method. A 500 bp paired-end sequencing library was subsequently constructed with the NEBNext Ultra DNA Library Prep Kit (NEB, Ipswich, MA, USA). High-throughput sequencing was then carried out on an Illumina NovaSeq 6000 system (BI-OZERON Co., Ltd., Shanghai, China), which produced approximately 8 GB of raw data for globe fringerush, with a read configuration of 150 bp paired-end. The raw sequence data were deposited in the Genome Sequence Archive [69] in National Genomics Data Center [70] (https://ngdc.cncb.ac.cn/gsa, accessed on 17 November 2025).

### 4.2. Genome Assembly

Given the absence of prior genomic data for this species, a de novo assembly approach was employed for chloroplast genome reconstruction [71]. Initial assembly was performed using NOVOPlasty (https://github.com/ndierckx/NOVOPlasty, accessed on 7 April 2025), incorporating closely related chloroplast genomes as references, which yielded two candidate circular contigs. The contig demonstrating the highest sequence similarity to known chloroplast DNA was chosen for further processing. To enhance assembly accuracy, potential chloroplast-derived reads were identified from the total Illumina sequencing pool via BLAST searches (https://blast.ncbi.nlm.nih.gov/Blast.cgi, accessed on 7 April 2025) against both the NOVOPlasty-generated contig and available chloroplast genomes of related taxa. These filtered reads were then reassembled de novo using SPAdes v3.13.0. The preliminary NOVOPlasty assembly was subsequently refined by integrating scaffolds generated by SPAdes, followed by alignment of the original clean reads with BWA and base correction using Pilon v1.22. The resulting sequence was finally reordered and oriented according to a reference chloroplast genome to produce the complete, finalized genome assembly.

### 4.3. Genome Component Analysis

The annotation of protein-coding genes, tRNAs, and rRNAs in the chloroplast genome of globe fringerush was carried out with GeSeq (https://chlorobox.mpimp-golm.mpg.de/geseq.html, accessed on 7 April 2025). Parameter settings included a protein search identity threshold of 60 [72] and 35 for rRNA, tRNA, and DNA search identity [73], with tRNA prediction supplemented by tRNAscan-SE v2.0.7. A high-confidence gene set was generated by eliminating redundancies from the initially predicted genes, followed by manual inspection and adjustment of gene start/stop codons and exon-intron junctions. Additionally, the nucleotide composition of the chloroplast genome was analyzed, and the spatial distribution of genes across the LSC, SSC, and IR regions was documented. Finally, functional categorization of all annotated genes was summarized.

### 4.4. Gene Function Annotation and Classification Analysis

The protein sequences derived from chloroplast genes were queried against several established protein databases via BLASTP (e-value < 1 × 10^−5^) [74]. To maintain biological relevance, when multiple alignment results were available for a single sequence, only the top hit was retained for subsequent analysis. The databases employed in this study comprised the Non-Redundant Protein Sequence Database (NR) (http://www.ncbi.nlm.nih.gov/, accessed on 7 April 2025), Swiss-Prot (http://www.ebi.ac.uk/uniprot, accessed on 7 April 2025), Clusters of Orthologous Groups (COG), Kyoto Encyclopedia of Genes and Genomes (KEGG) (http://www.genome.jp/kegg/, accessed on 7 April 2025), and Gene Ontology (GO) (http://geneontology.org/, accessed on 7 April 2025). Functional annotations for the coding genes of globe fringerush were subsequently obtained by aligning their amino acid sequences against these databases.

### 4.5. Contraction and Expansion Analysis of Inverted Repeats Regions

In conjunction with the newly sequenced chloroplast genome of *Panicum bisulcatum*, another 12 plants of the genus *Panicum*, and four common Poaceae plants in paddy fields (Supplementary Table S3) were retrieved from NCBI for comparative analysis of inverted repeat (IR) regions. The organizational structure of each genome—comprising the large single-copy (LSC), small single-copy (SSC), and two IR regions—was systematically examined. We further assessed the consequences of IR boundary shifts, such as variations in gene copy number and potential pseudogenization resulting from IR expansion or contraction [75]. Genes located at or spanning these junctions were identified and examined with respect to their functional annotation, sequence length, and positional relationship to the IR-SC boundaries.

### 4.6. Phylogenetic Analysis

Alongside the newly assembled chloroplast genome of *Panicum bisulcatum*, an additional twelve *Panicum* species and four commonly occurring Poaceae plants in paddy fields (Supplementary Table S3) were obtained from the NCBI database for phylogenetic reconstruction. Multiple sequence alignment was performed with ClustalW (v2.0.12) under default parameters. The optimal nucleotide substitution model was selected using the Akaike information criterion [76]. Phylogenetic inference based on the maximum likelihood (ML) method was carried out with PhyML v3.0 (http://www.atgc-montpellier.fr/phyml/, accessed on 7 April 2025), with branch support evaluated through 1000 bootstrap replicates [77,78]. Additionally, a Bayesian inference analysis was conducted using MrBayes 3.1.2, adopting the methodology outlined by Wu et al. [79]. The phylogenetic tree was rooted using *Leersia japonica* as the outgroup, which belongs to the genus *Leersia* and is established to be phylogenetically outside the clade containing the genus *Panicum* and its close relatives within Panicoideae.

### 4.7. Single Nucleotide Polymorphism (SNP) Analysis

The chloroplast genome sequences of *Panicum bisulcatum* (as reference) and its closest relative, *P. incomtum*, identified through prior phylogenetic and IR boundary analyses, were subjected to whole-genome alignment using MUMmer software (http://mummer.sourceforge.net/, accessed 7 April 2025). An initial screening was conducted to identify putative single nucleotide polymorphism (SNP) sites. To validate each candidate SNP, 100 bp flanking regions centered on the reference SNP position were extracted and realigned against the assembled genome of *P. incomtum* using BLAT v35 (http://hgdownload.soe.ucsc.edu/admin/exe/linux.x86_64/blat/, accessed 7 April 2025). Candidate sites with alignment lengths shorter than 101 bp were discarded as unreliable, and those aligning to multiple genomic locations were filtered out as repetitive elements. This stringent process ensured that only high-confidence SNPs were retained for further analysis.

## 5. Conclusions

This study provides the first comprehensive analysis of the chloroplast genome of *Panicum bisulcatum*, offering detailed insights into its structural features, repetitive elements, gene content, IR boundary dynamics, and evolutionary history. The genomic resources and molecular markers (SSRs, LRs, SNPs) identified here will support future studies in species identification, phylogenetic reconstruction, population genetics, and biogeography. From an applied perspective, a deeper genetic understanding of this weedy species can inform the development of more specific molecular tools for monitoring its distribution and spread. Furthermore, these genomic resources establish a crucial foundation for future research on the evolution, ecology, and management of this agriculturally important weed species.

## Figures and Tables

**Table 1:** List of genes encoded by chloroplast genome of *Panicum bisulcatum*.

Category	Gene Groups	Gene Name
Photosynthesis	Subunits_of_photosystem_I	*psaA*, *psaB*, *psaC*, *psaI*, *psaJ*
	Subunits_of_photosystem_II	*pbf1*, *psbA*, *psbB*, *psbC*, *psbD*, *psbE*, *psbF*, *psbH*, *psbI*, *psbJ*, *psbK*, *psbL*, *psbM*, *psbT*, *psbZ*
	Subunits_of_NADH_dehydrogenase	*ndhA*, *ndhB*, *ndhC*, *ndhD*, *ndhE*, *ndhF*, *ndhG*, *ndhH*, *ndhI*, *ndhJ*, *ndhK*
	Subunits_of_cytochrome_b/f_complex	*petA*, *petB*, *petD*, *petG*, *petL*, *petN*
	Subunits_of_ATP_synthase	*atpA*, *atpB*, *atpE*, *atpF*, *atpF*, *atpH*, *atpI*
	Large_subunit_of_Rubisco	*rbcL*
Genetic system	Large_subunits_of_ribosome	*rpl14*, *rpl16*, *rpl20*, *rpl22*, *rpl23*, *rpl32*, *rpl33*, *rpl36*
	Small_subunits_of_ribosome	*rps11*, *rps12*, *rps14*, *rps15*, *rps16*, *rps18*, *rps19*, *rps2*, *rps3*, *rps4*, *rps7*, *rps8*
	DNA-dependent_RNA_polymerase	*rpoA*, *rpoB*, *rpoC1*, *rpoC2*
	Ribosomal_RNAs	*rrn16*, *rrn23*, *rrn4.5*, *rrn5*
	Transfer_RNAs	*trnA-UGC*, *trnC-GCA*, *trnD-GUC*, *trnE-UUC*, *trnF-GAA*, *trnG-GCC*, *trnG-UCC*, *trnH-GUG*, *trnI-CAU*, *trnI-GAU*, *trnK-UUU*, *trnL-CAA*, *trnL-UAA*, *trnL-UAG*, *trnM-CAU*, *trnN-GUU*, *trnP-UGG*, *trnQ-UUG*, *trnR-ACG*, *trnR-UCU*, *trnS-GCU*, *trnS-GGA*, *trnS-UGA*, *trnT-GGU*, *trnT-UGU*, *trnV-GAC*, *trnV-UAC*, *trnW-CCA*, *trnY-GUA*, *trnfM-CAU*
Other genes	Maturase	*matK*
	Protease	*clpP1*
	Envelope_membrane_protein	*cemA*
	Acetyl-CoA_carboxylase	
	C-type_cytochrome_synthesis_gene	*ccsA*
	Translation_initiation_factor	*infA*
	protochlorophillide_reductase_subunit	
Unknown Genes	Proteins_of_unknown_function	*ycf2*, *ycf3*, *ycf4*, *ycf68*

**Table 2:** Single nucleotide polymorphisms (SNPs) in the *Panicum incomtum* chloroplast genome compared to the *P. bisulcatum* chloroplast genome.

Species	Start	Stop	Synonymous	Nonsynonymous	CDS	Intergenic	Total SNPs
*Panicum incomtum*	2	8	2000	1252	3281	1378	4659

## Data Availability

The data presented in this study are openly available in [China National Center for Bioinformation] at [https://ngdc.cncb.ac.cn/gsub/submit/gsa/subCRA054879/finishedOverview, accessed on 17 November 2025], reference number [PRJCA051150].

## Supplementary Materials

The following supporting information can be downloaded at: https://www.mdpi.com/article/10.3390/ijms27010135/s1.
