# Supplementary figures and images for: The Complete Chloroplast Genome and the Phylogenetic Analysis of Panicum bisulcatum (Thumb.) (Poaceae)
Source: Int J Mol Sci. 2025 Dec 22;27(1):135. doi: 10.3390/ijms27010135 (PMC12785542; doi:10.3390/ijms27010135)

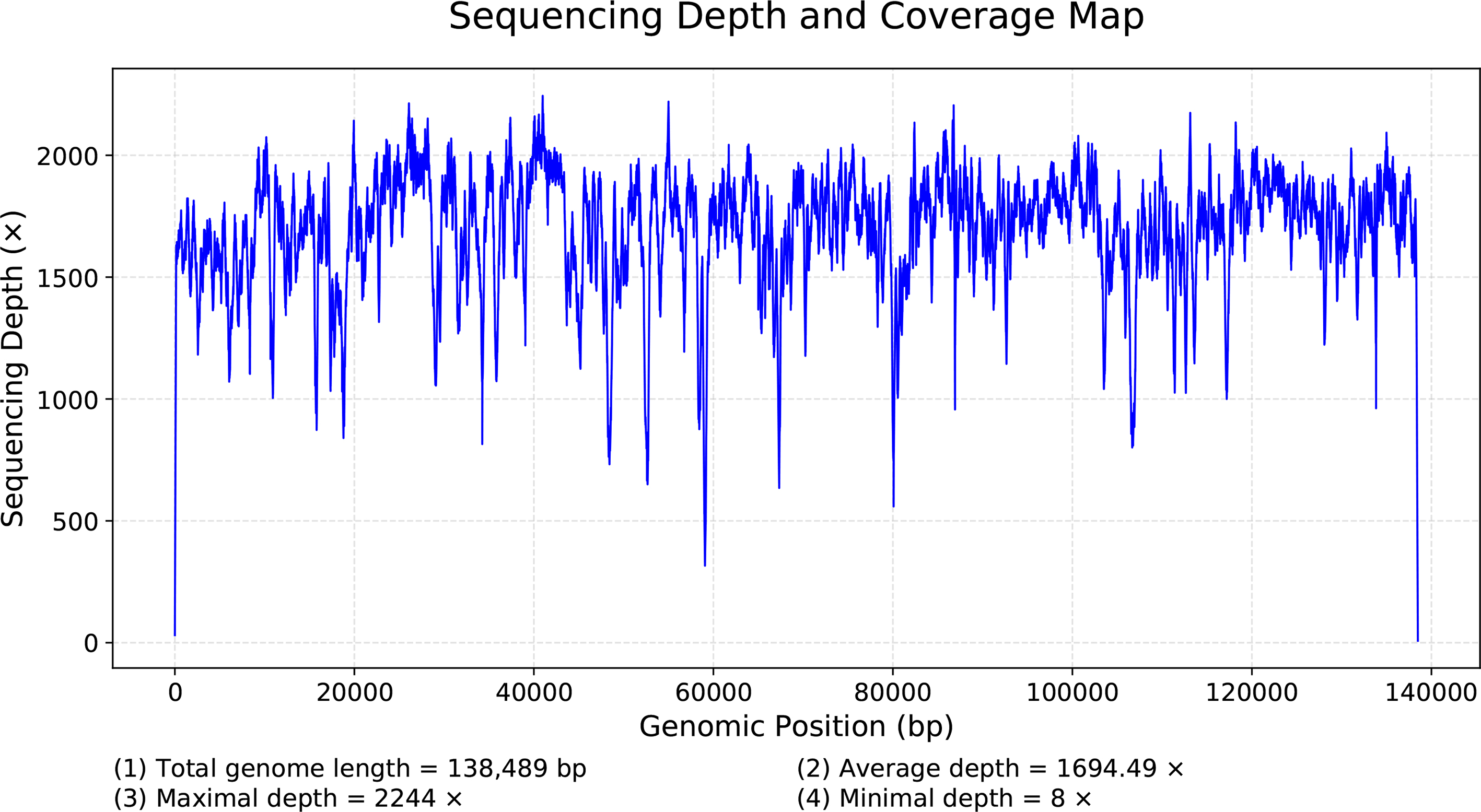

Supplement: Supplementary file 1 [file ijms-27-00135-s001.zip › Supplementary Figure S1 The sequencing depth and coverage map of the Panicum bisulcatum sample.jpg]
